# Perineural spread in head-and-neck malignancies: Imaging findings – An updated literature review

**DOI:** 10.17305/bjbms.2021.5897

**Published:** 2021-06-29

**Authors:** Olga Medvedev, Mihaela Hedesiu, Anca Ciurea, Manuela Lenghel, Horatiu Rotar, Cristian Dinu, Rares Roman, Dragos Termure, Csaba Csutak

**Affiliations:** 1 Department of Radiology, County Clinical Emergency Hospital Cluj, Cluj-Napoca, Romania; 2 Department of Oral and Maxillofacial Radiology, University of Medicine and Pharmacy Cluj-Napoca, Romania; 3 Department of Radiology, Faculty of Medicine, “Iuliu Hațieganu” University of Medicine and Pharmacy, Cluj-Napoca, Romania; 4 Department of Cranio-Maxillofacial Surgery, “Iuliu Hațieganu” University of Medicine and Pharmacy, Cluj-Napoca, Romania; 5 Department of Maxillofacial Surgery and Implantology, Faculty of Dentistry, “Iuliu Hațieganu” University of Medicine and Pharmacy, Cluj-Napoca, Romania; 6 Department of Cranio-Maxillofacial Surgery, Faculty of Dentistry, “Iuliu Hațieganu” University of Medicine and Pharmacy, Cluj-Napoca, Romania

**Keywords:** Head-and-neck tumors, perineural invasion, cranial nerves, neuroanatomy imaging methods

## Abstract

Perineural spread (PNS) represents the tumor’s ability to disseminate along nerves. The aim of this article is to review the relevant literature about the PNS in head-and-neck tumors. The important information for imaging analysis is summarized in a diagnostic flowchart. The pathogenesis, clinical signs, prognostic importance, and technical considerations for computer tomography and magnetic resonance imaging are briefly discussed. The anatomical pathways of the cranial nerves (CNs) and the main checkpoints are synthesized. Most commonly affected nerves are the trigeminal and facial, although any of the CNs may be involved. The described imaging features represent important clues for an optimal differential diagnosis. PNS worsens the prognosis and significantly changes the treatment, thus radiologists should be aware of this entity and be able to find it on imaging in the appropriate clinical context.

## INTRODUCTION

Head-and-neck (H and N) malignancies comprise a considerable variety of histologic subtypes, the most common type being the squamous cell carcinoma (SCC). Among the ways by which H and N tumors are disseminated, it is well recognized that the neoplastic cells can advance along the neural sheath by a process called perineural tumor spread. Although it was a theme of many research works, there is still a lot of confusion about the precise definition [1], and therefore about the true incidence. The presence of perineural spread (PNS) implies worsening of the prognosis and change in treatment and it may occur even in the absence of hematogenous or lymphatic metastasis [2]. There are several crucial points that allow PNS imaging diagnosis: adequate imaging techniques, familiarity with the commonly associated types of cancer, good knowledge of the common nerve routes, and imaging appearance of PNS.

This article presents a diagnostic flowchart based on the literature review, to facilitate the observation of the PNS on the computed tomography (CT) and magnetic resonance imaging (MRI).

## UNDERSTANDING THE TERMINOLOGY: PNS VERSUS PERINEURAL INVASION (PNI)

Up to date, the literature is still inconclusive and somewhat confusing regarding the precise definition of PNI and PNS. Batsakis et al. [3] defined PNI in 1985 as tumor cell invasion “in, around, and through nerves,” a broad definition that leaves space for more detailed clarification. In their review, Liebig et al. [2] are in accordance with Batsakis, but offering a more precise description: “The finding of tumor cells within any of the three layers [epi-, peri-, or endoneurium] of the nerve sheath or tumor in close proximity to nerve and involving ≥33% of its circumference,” a definition widely accepted and cited by many other authors [4-8].

In radiology literature, two different terms describe perineural growth: PNI as tumor cells infiltrating small unnamed nerves, which can only be seen microscopically, but not radiologically, often limited to the main tumor mass [9], and PNS, meaning the gross tumor spread along a larger, typically named nerve, at least in part distinct from the main tumor mass and evident on imaging studies [7,10-13]. There is not an exact definition of the transition from PNI to PNS, but the last is evident on MRI and may show clinical manifestations corresponding to the involved nerve [12]. A radiological challenge is deciding if the cranial nerve (CN) situated within or immediately adjacent to a tumor can be termed as PNS, though many specialists consider that PNS refers to “tumor selectively traveling along a nerve away from a primary lesion” and “separate from the main bulk of the tumor” [9,14,15]

The trigeminal nerve (CN V) and the facial nerve (CN VII) are the most affected nerves mainly because of their extensive innervated territory, though virtually any CN and its branches can constitute a route for the PNS [16].

## PATHOGENESIS OF PNS. THE MOST COMMON HEAD AND NECK CANCERS ASSOCIATED WITH PNS

The concept of PNI was first brought into discussion in 1835 (Cruveilheir) and 1862 (Neumann) [17] and suffered several changes in the understanding of its pathogenesis: first thought to be a lymphatic spread of tumor to the nerves, then stipulated that the nerve sheath is a low resistance path for tumor spread. These theories have now been mistrusted and the new research shows evidence that the signaling between the nerves, the invading tumor cells, and stromal elements composes the main mechanism behind PNI, with a reciprocal growth interaction occurring between nerves and tumors [2,12]. This is possible due to various factors produced by the neoplastic cells and the local microenvironment (neurotrophins such as NGF, BDNF, and NT-3) [2,5,18].

Regarding H and N cancers, the incidence is estimated around 27–82% of H and N mucosal or cutaneous SCCa [5] and approximately 31–96% in case of adenoid cystic carcinoma (ACC) [5,19]. Regarding ACC, even if it represents only 1-3% of H and N cancers, it has the highest relative incidence of PNI [20]. PNI is also frequently found in patients with salivary duct carcinoma, polymorphous low-grade adenocarcinomas [5], cutaneous malignancies, desmoplastic melanoma, myeloma, lymphoma, and leukemia [7,21].

## CLINICAL SIGNS ASSOCIATED WITH PNS

Up to 40% of patients with perineural involvement are asymptomatic or have non-specific symptoms [5,9-11]. However, there are some “red flags” that should alert the physician and the radiologist to raise suspicion of PNS in an appropriate clinical context, as the unexplained neurologic symptoms may appear well before the PNS is apparent on imaging. These correspond to the alteration of either sensory or motor function of the specific affected CN in the respective innervation territory, and include pain, paresthesias, numbness, formication, and motor denervation weakness. As the most common CNs involved are the CN V and CN VII, the weakness of the mastication muscles (mandibular division V3) or facial expression (CN VII) is expected, which evolves to muscle atrophy in chronic cases. Furthermore, there may be ipsilateral loss of sensation: anesthesia of forehead and absent corneal reflex when ophthalmic nerve (V1) is affected; anesthesia of the midface for maxillary nerve (V2); anesthesia of the chin, lower lip, and anterior 2/3 of the tongue for V3 [22].

If PNS reaches the lower CN, it can manifest as following: dysphagia, absent gag reflex, and uvula deviation (CN IX, X); hoarseness caused by vocal cord paralysis (CN X); pain and weakness of shoulder and/or neck movement, shoulder drooping and lateral displacement of the scapula (CN XI); and paralysis, fasciculation, and atrophy of the tongue muscles (CN XII) [22].

Attention must be paid to the differential diagnosis with trigeminal neuralgia and idiopathic facial paralysis (IFP or Bell’s palsy), both being the reason for PNS misdiagnosis. Trigeminal neuralgia causes episodic, lancinating, and triggerable facial pain with free intervals, whereas CN V neuropathy in PNS presents with constant, unilateral, and mild facial pain with strong sensory loss. The IFP is a unilateral weakness involving all peripheral branches of the facial nerve, gradually resolving overtime, with complete recovery of 70% of all patients [23]. In contrast, slowly progressive (>3 weeks) or recurrent paralysis, facial hyperkinesia, pain, and other CN involvement indicate another cause and one must think of potential PNS [5], as facial paralysis of neoplastic origin constitutes about 5% of all cases [23]. Furthermore, a sudden onset of facial paralysis does not guarantee the definite exclusion of tumoral cause [23].

Rarely, PNS may be attested clinically and by imaging before the primary cancer has manifested itself, as it may happen, for example, in clinically silent submucosal slow-growing tumor (ACC) originating in the palate [10].

Sometimes, the presence of clinical symptoms of PNS without any signs on imaging studies may suggest a microscopic PNI [24].

## IMPORTANCE OF PNS IN THE TREATMENT PLANNING AND PROGNOSIS

To apply a correct radiation procedure and/or surgery, the oncology team may have to make a decision without histopathology confirmation, because PNS usually appears in anatomic areas unapproachable for biopsy, therefore, radiology plays a crucial role in detecting this condition [25,26].

PNI/PNS is considered to be an adverse prognostic factor in H and N cancers of virtually all sites, associated with poor local and regional disease control, probability of locoregional and distant metastasis, disease recurrence, and decreased survival rate [27-29].

The accuracy and frequency of PNI reporting is questionable, because there is variation in the biopsy technique, method of detection, small number of cases analyzed, insufficient follow-up (due to natural history), as well as no unique standardized accepted definition [19,30]. Referring to imaging results, the statistics is controversial, with variations and contradictories in the studies about the prognostic significance [5,30].

Most H and N oncologists consider the presence of PNI at least a relative indication for post-operative radiotherapy in resected cutaneous and mucosal SCCa and aggressive salivary gland tumors [5], as well as in any primary tumor with clinical or radiological signs of PNS [31]. The detection of PNS is critical for careful pre-operative planning, as well as for radiation planning target volume [5,31,32]. For example, facial nerve-sparing surgery in some parotid tumors may be attempted and the surgeon must know if the facial nerve is intact [20]. When the tumor spread is found cranially to the geniculate or Gasserian ganglion, the case is considered as inoperable [33].

Failure to recognize the PNS goes with high risk of morbidity from cancer progression; the recurrence of PNS is rarely resectable and the repeated irradiation has greater toxic adverse effect in the detriment of fewer benefits when compared with the initial early treatment [31,34].

## DIAGNOSTIC APPROACH BASED ON THE LITERATURE REVIEW

**FIGURE 1:**
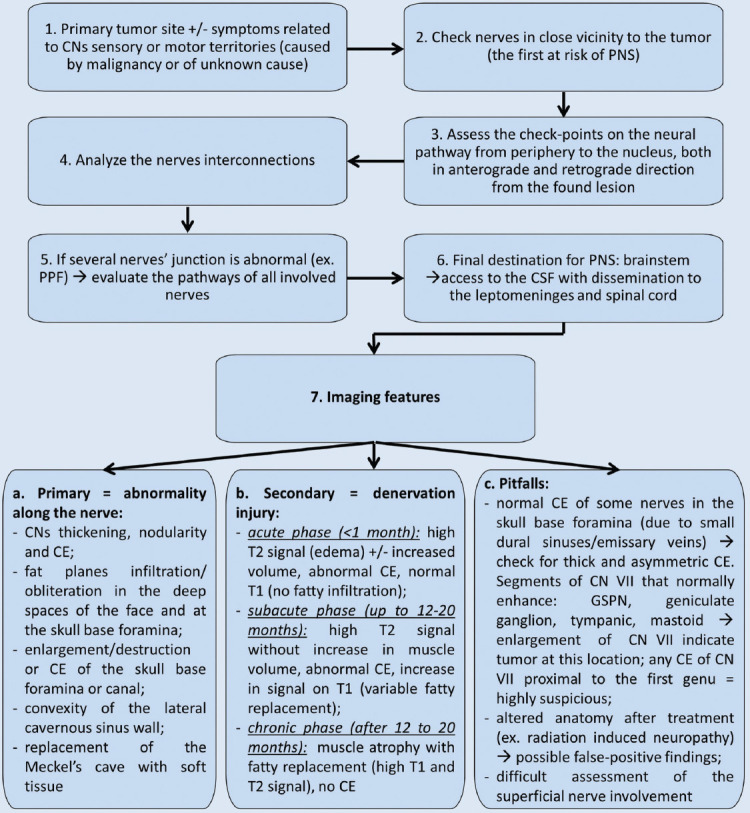
The diagram presents the imaging diagnostic steps for perineural spread (PNS) in head-and-neck cancers. CNs: Cranial nerves; PPF: Pterygopalatine fossa; CSF: Cerebrospinal fluid; CE: Contrast enhancement; GSPN: Greater superficial petrosal nerve.

Based on the available literature, a diagnostic algorithm is proposed in Figure 1. The success of a correct diagnosis is assured most importantly by the complete clinical information, which determines the direction of search, as well as the comprehensive knowledge of CNs anatomy which constitutes the road map to the neural lesion.

### CNs relevant anatomy for PNS “chasing”

The tumoral cells can invade in retrograde or centripetal direction from the primary tumor proximally toward the brain stem, as well as anterograde or centrifugal once tumor reaches main connection points (pterygopalatine fossa – PPF, Meckel’s cave) spreading to the periphery [9-11,16]. Another spreading feature is the “skip lesion” which means an uninvolved segment on imaging along the affected nerve between the primary tumor and metastatic site, while microscopically the areas of nerve invasion can be continuous [9,10,26] and could be explained by the amount of tumor burden along a nerve’s route [9]. Thus, the radiologist must always inspect the entire nerve.

**FIGURE 2:**
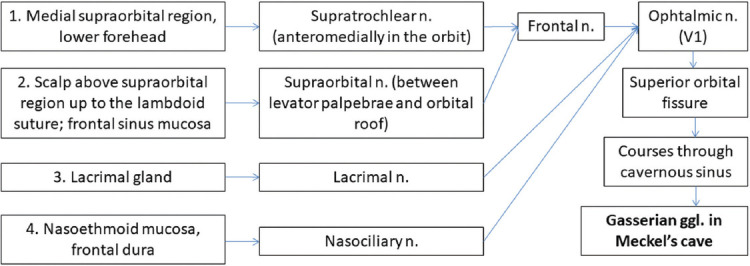
Potential PNS routes along ophthalmic nerve (V1), starting from primary tumor in a retrograde manner (blue arrows). Pathways 3 and 4 are less commonly involved [7,10,11,35]. n: Nerve; ggl: Ganglion.

**FIGURE 3:**
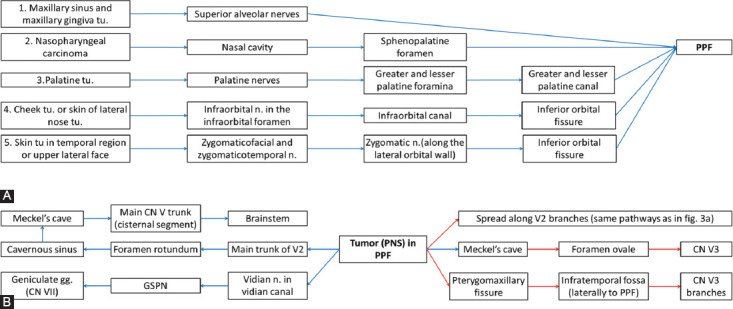
(A) Potential PNS routes along maxillary nerve (V2), commonly aiming pterygopalatine fossa (PPF). (B) From PPF, the tumor (tu.) can spread either in more common retrograde (blue arrows) or in anterograde (red arrows) fashion [7,10,11,35]. n: Nerve; ggl: Ganglion; GSPN: Great superficial petrosal nerve.

**FIGURE 4:**
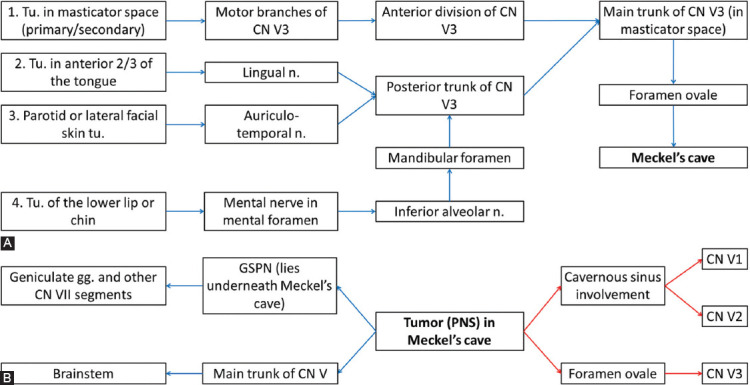
(A) Potential PNS routes along mandibular nerve (V3) toward Meckel’s cave; (B) From Meckel’s cave, the malignant cells can spread either retrograde (blue arrows) or antegrade (red arrows), reaching even facial nerve (CN VII) through greater superficial petrosal nerve (GSPN) [7,10,11,35]. Tu: Tumor; n: Nerve; gg: Ganglion.

**FIGURE 5:**
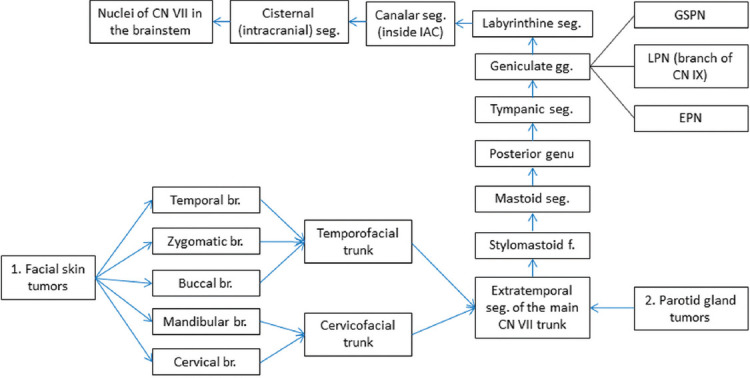
Possible routes for PNS along the facial nerve (CN VII), blue arrows = retrograde spread. The geniculate ganglion (gg) has four branches (br.): Tympanic, great superficial petrosal nerve (GSPN), lesser petrosal nerve (LPN), and external petrosal nerve (EPN), of which GSPN constitutes an important retrograde tumoral route due to connections with the maxillary nerve [7,10,11,35]. seg: Segment, f: Foramen, IAC: Internal auditory canal.

The main pathways along the CNs for the PNS are presented in Figures 2-5 [7,10,11,35].

**FIGURE 6:**
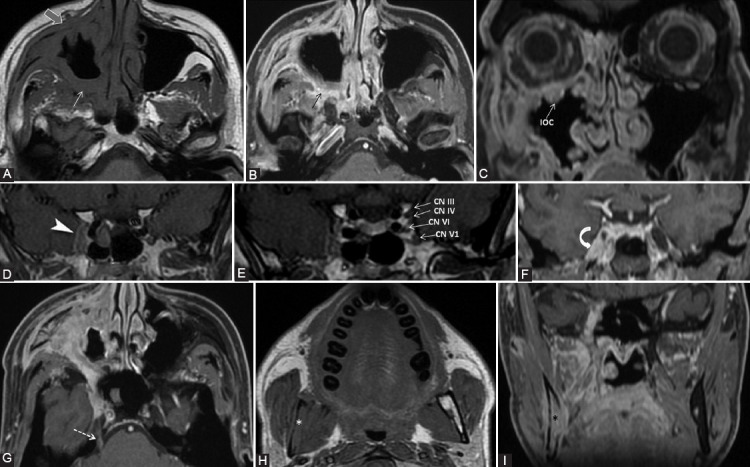
A 39-year-old male diagnosed with adenoid cystic carcinoma of maxillary sinus, with multiple extensions. Clinically: Right abducens nerve palsy, diplopia, right infraorbital anesthesia. Axial T1-weighted (A, H), axial (B, G), and coronal (C, F, I) contrast-enhanced (CE) 3D T1 FSPGR; coronal contrast enhanced in Phase T1 FSE IDEAL (D, E) images. The probable first point of PNS is PPF with fat obliteration (white arrow) and CE (black arrow), where the V2 nerve is involved. In an anterograde direction, the tumor reaches the right infraorbital nerve, demonstrated by infraorbital canal enlargement with CE (IOC) and preantral fat obliteration (gray arrow). Following the main V2 trunk, the tumor reaches the cavernous sinus which appears larger and shows CE (arrowhead). Note that in CN III, IV, V1, and V2 are situated in the lateral wall of the cavernous sinus, the cavernous segment of CN VI is located medially to the CN V1, implying the tumoral involvement of the mentioned nerves. There is Meckel’s cave CE (curved arrow) and the tumor traveled back up to the main trigeminal trunk (cisternal segment, dashed arrow). Notice the right mandibular fat replacement (white *), associated with mandibular CE (black *), and cortical disruption, meaning an anterograde PNS from the Meckel’s cave to the inferior alveolar nerve (branch of V3).

When a suspect lesion is encountered at one of the nerves’ “meeting point” (PPF, Meckel’s cave, cavernous sinus), the pathways of all the CNs that pass through should be inspected [9]. An example of such complex involvement of multiple CNs is illustrated in Figure 6.

**FIGURE 7:**
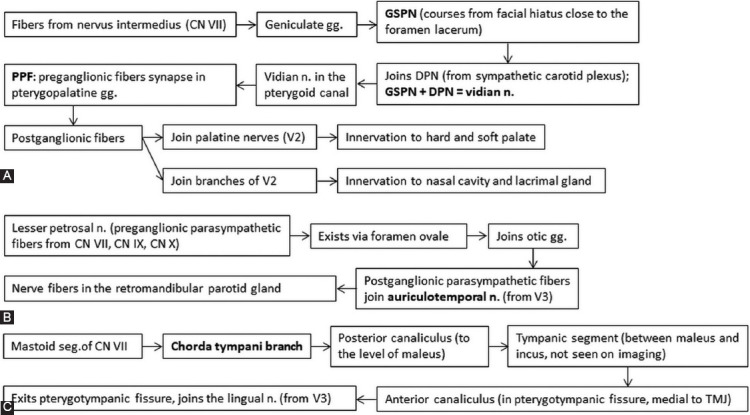
Interconnections between trigeminal (CN V) and facial nerve (CN VII). Figure (A) shows how the great superficial petrosal nerve (GSPN) is formed and connects with branches of maxillary nerve (V2). Any tumor that reaches the pterygopalatine fossa (PPF) can evolve to PNS along the GSPN and other segments of CN VII. Also, because the GSPN passes underneath the Meckel’s cave, any tumor that reaches the trigeminal cavity can easily involve the GSPN. Diagram (B) indicates the connection between auriculotemporal nerves (branch of mandibular nerve V3, passes into the parotid gland along the posterior margin of the mandibular ramus) with the CN VII through lesser petrosal nerve. Figure (c) depicts the course of chorda tympani nerve branching off the mastoid segment of CN VII until it joins the lingual nerve after exiting the skull through the petrotympanic fissure [7,10,11,35]. DPN: Deep petrosal nerve; gg: Ganglion; n: Nerve, TMJ: Temporomandibular joint.

There are important nerve interconnections used by tumoral cells to disseminate. Most often recognized on imaging are the greater superficial petrosal nerve (GSPN) linking V2 with the geniculate ganglion of CN VII (schematically represented in the Figure 7a) and the auriculotemporal nerve (ATN) that communicates with CN VII within the parotid gland (Figure 7b) [10,36,37].

Furthermore, the chorda tympani nerve (from CN VII) joins the lingual nerve (from V3) as represented in Figure 7c.

### Important anatomical checkpoints along the nerves’ route

The possible primary tumor site corresponding to the innervated territory, as well as imaging checkpoints along the nerves’ course, which have to be assessed on the imaging, are synthesized in Tables 1-3. The juxtaforaminal fat pads are important clues to be analyzed.

**TABLE 1:**
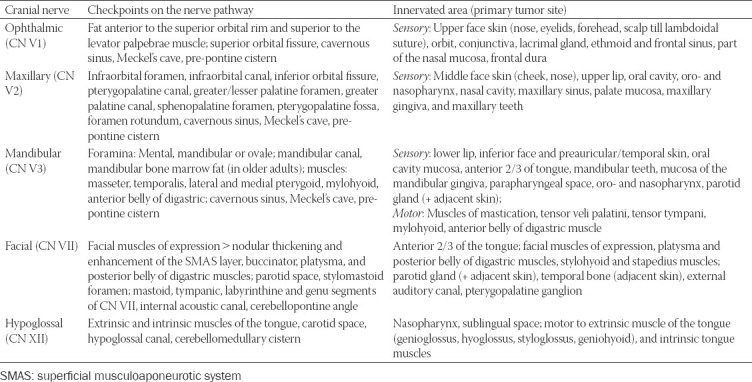
Check-points along the nerve’s route where MRI features indicative for perineural spread (PNS) in commonly involved CNs can be found. The table also contains the innervated area supplied by each cranial nerve (CN), making it the first nerve at risk according to the location of the primary tumor [7,10,11,22,35]

**TABLE 2:**
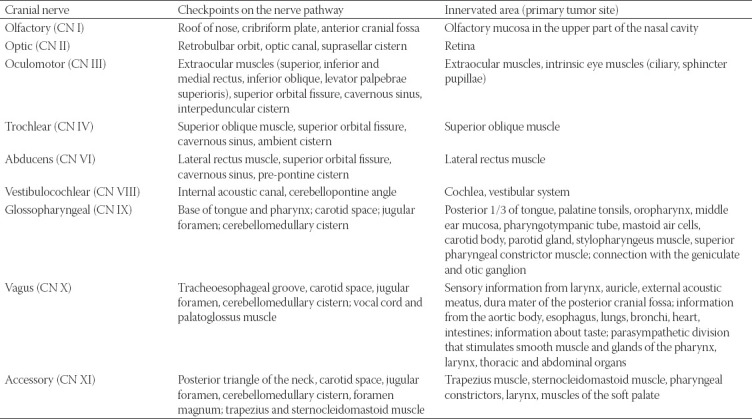
Check-points for PNS along the nerve’s pathway for the CNs rarely involved in the perineural tumor spread [7,10,11,22,35]]

**TABLE 3:**
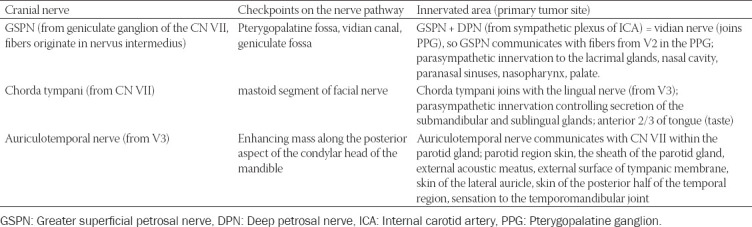
Important nerve interconnections between the peripheral branches of CN V and CN VII with the specific check-points where features of PNS can be traced [7,10,11,22,35]

**FIGURE 8:**
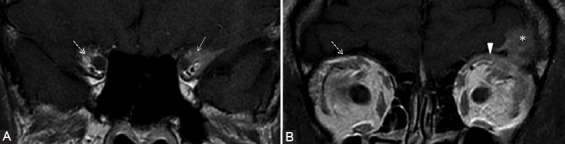
Patient with the left lacrimal gland adenoid cystic carcinoma operated for 6 years, presented for numbness of the forehead. There is tumoral relapse at the level of superior rectus and levator palpebrae muscle. The coronal contrast-enhanced T1-weighted FSE (A, B) demonstrates CE of the fat pad around the CN V1 in the orbital apex (arrow) and fat pad obliteration superior to the levator palpebrae muscle (arrowhead) in the expected location of the V1 branches, compared to their normal appearance on the right side (dashed arrows). The findings are suggestive of PNS along V1. The left frontal cerebral metastasis (*) is also noticed.

Although PNS can occur from any potential neoplasm in the innervated territory of the CNs, there are more commonly encountered situations. The majority of PNS along V1 occur from cutaneous malignant neoplasms such as SCCa or melanoma of forehead, anterior scalp, upper eyelid, and affecting the supraorbital or supratrochlear nerve [38]. Respectively, the radiologist should check up the fat pad in the orbital apex, the fat tissue along the orbital roof upper to the superior rectus and above levator palpebrae superioris muscles (Figure 8), as well as anterior to the superior orbital rim, all visible on CT and MRI.

**FIGURE 9:**
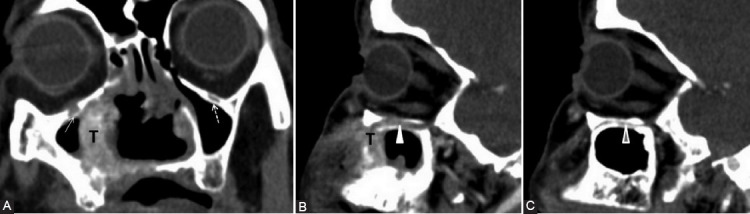
Nasal squamous cell carcinoma (T) with maxillary extension. Coronal post-contrast computed tomography image (A) shows enlargement and contrast enhancement of the right infraorbital nerve in its corresponding foramen (arrow). The enlarged infraorbital canal (white arrowhead) shown on the sagittal view (B, C) is a pathway for the PNS toward the inferior orbital fissure and then the pterygopalatine fossa. Notice the normal left infraorbital foramen fat pad (dashed arrow) and canal (gray arrowhead).

**FIGURE 10:**
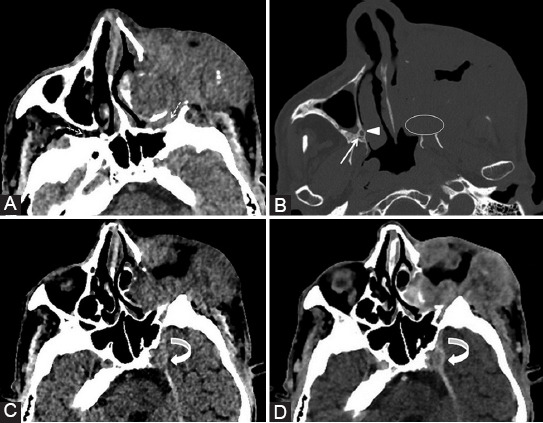
The left maxillary squamous cell carcinoma with orbital extension. Axial computed tomography (CT) image (A) reveals the obliteration of PPF fat pad on the left compared to the right side (dashed arrows). Bone window axial CT scan (B) shows complete involvement of the left palatine foramina (ellipse) versus the right greater (arrowhead) and lesser (arrow) foramina. Axial CT before (C) and post-contrast (D) administration demonstrates left Meckel’s cave tissue effacement and contrast enhancement at this level (curved arrow).

Common tumoral sites that can cause PNS along V2 branches are neoplasms of oral cavity or oropharynx mucosa (especially hard and soft palate), sinonasal tract (Figure 9), and facial skin cutaneous cancers. For V2 spread there are 3 important factors: conspicuous fat in the PPF (Figure 10), the retroantral and preantral fat (at the opening of the infraorbital foramen), as well as the fat near to foramen rotundum.

**FIGURE 11:**
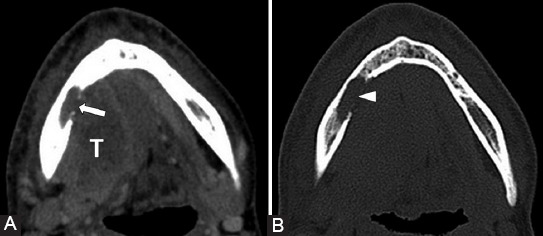
Squamous cell carcinoma (T) of the tongue base and oral floor. Soft-tissue (A) and bone window (B) axial computed tomography images demonstrate tumor invasion in the mandibular body on the right (arrow) with bone erosion into the alveolar canal (arrowhead), suggesting PNS along V3.

The motor branches of V3 may serve as a metastatic route in case of tumors in the masticator space, nasopharynx, oropharynx, or a retrograde spread from the inferior alveolar nerve (Figure 11) [39]. Considering V3, there is the fat pad anterior to mental foramen, at the mandibular and ovale foramen, as well as the mandibular bone marrow fat that surrounds the inferior alveolar canal which needs to be assessed (Figure 6).

**FIGURE 12:**
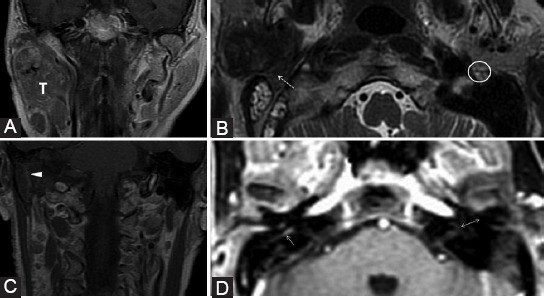
Parotid adenocarcinoma (T) of superficial and profound lobes, extending in the area of facial nerve distribution; the patient presented with the right peripheral facial palsy. Axial T2-weighted image (B) shows right stylomastoid foramen fat effacement (dashed arrow) versus the left side (ellipse). Coronal contrast-enhanced T1-weighted images (A, C) reveal retrograde PNS along the mastoid segment of the right CN VII that shows CE (arrowhead). Dynamic contrast enhancement sequence (D) demonstrates possible upper involvement of the CN VII as there appears asymmetric CE of the right tympanic segment (arrow) when compared to the left side (double arrow).

The mastoid segment and the GSPN are the most affected segments of CN VII in case of PNS. The primary parotid tumors or the neoplasms invading the gland are responsible for nerve involvement (Figure 12). At the exit point of the CN VII from the stylomastoid foramen, a visible fat pad can be noticed and must always be assessed [40].

The PNS along the hypoglossal nerve arises more often from a nasopharyngeal carcinoma that spreads to the retrostyloid compartment of the parapharyngeal space [39].

### Technical considerations

CT and MRI complete each other and are useful to guide the diagnosis. CT can spot the PNS at later stages than MRI, when there are voluminous tissue masses with foraminal erosions and widening. CT studies may be useful for biopsy guidance, as well as in patients with various implants that are incompatible with MRI.

However, MRI provides a superior soft-tissue contrast compared to CT, so it is considered best suited for detecting PNS and for identifying the anatomic extent of spread [5,16,41]. Nemzek et al. concluded that 1.5 T MRI has 95% sensitivity for PNS detection, but cannot always delineate the true extent of the tumoral spread, as the microscopic foci are only determined histologically [9]. Furthermore, Baulch et al. in a study about 3T MRI evaluation of large nerves PNS determined a sensitivity of 95% and a specificity of 84% for the 3T MRI [33]. In a recent study, Schroeder et al. concluded that 1.5 T MRI has a sensitivity of 62% and a specificity of 88%, for detecting PNS in a cohort of patients with H and N SCCa [42].

For small CNs, one must use high-resolution imaging with thin sections (maximum 3 mm) [8] and small field of view for both CT and MRI (no greater than 18 cm) [8,10]. It is mandatory to include a high-resolution bone algorithm for CT for assessing bone erosions (Figures 10 and 11). The three-dimensional (3D) isotropic sequences became routinely used, as they allow reformations in standard planes and along the course of an affected nerve, but due to longer acquisition time, it may cause motion artifacts with low-quality images. Thus, the protocol should include standard and 3D imaging [8]. Coronal, axial, and sagittal planes must all be reviewed, as some points of interest are better visualized on a particular plane (e.g. Meckel’s cave, foramen ovale, and V3 trunk on coronal image). Thereby, the coronal plane depicts better the CN I-VI, as they mainly traverse posteroanteriorly. The rest of CNs course are more mediolaterally and are better evaluated on the axial plane [35,43]. The imaging limits should be: on axial – from above the orbit’s roof down to the tip of the mandible; on coronal – from the globes anteriorly to the posterior margin of the temporal bones.

### Imaging protocols

There are different opinions regarding the best MRI protocol, the sequences listed in Table 4 are important to be included.

**TABLE 4:**
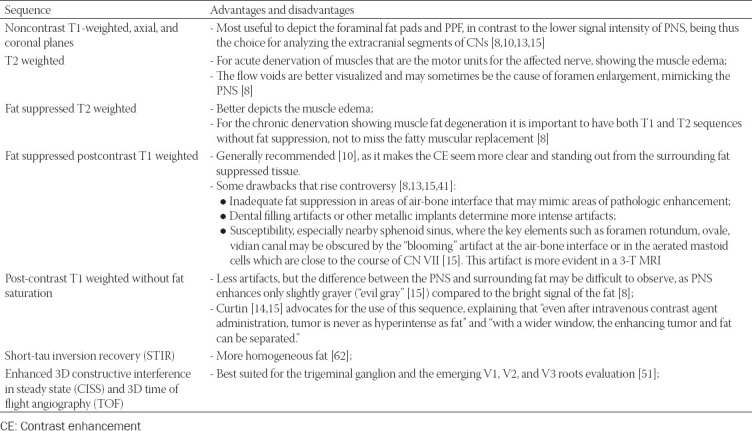
The most important MRI sequences that need to be included in the examination protocol intended for PNS evaluation [8,10,13,15,41,44-46]

In general, when imaging the CNs, the sequences should be adapted according to the CNs segments, as shown in Table 5, so these principles may also be adjusted to the study of PNS. Thus assessing the CNs, one should start from the nucleus (located in the brain for the CN I-II and in the brainstem for CN III-XII), then follow the cisternal segment where the nerve is surrounded by cerebrospinal fluid. Subsequently the pathway follows the vascular segment where the nerves are running through the venous plexuses including: CNs III-VI in the cavernous sinus to the superior orbital fissure, CN VII-VIII in the cerebellopontine angle and internal auditory canal, CN IX-XI in the jugular foramen, and CN XII in the hypoglossal canal. Finally, the CNs enter their extracranial part, where they are found in the soft tissues of the face and neck, mainly surrounded by fat [35].

**TABLE 5:**
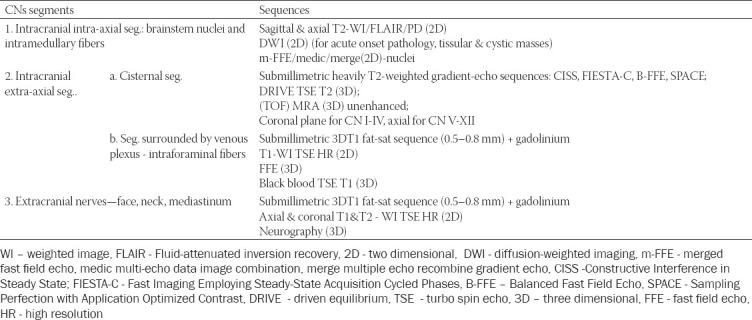
Variations of MRI sequences adjusted to the CNs regional anatomy (the suitable sequence depends on the tissue/fluid that is surrounding the nerve), used for imaging the CNs different pathologies (adapted from [35, 46, 47])

As newer imaging techniques are emerging, there may be an even more accurate visualization of CNs pathology in the future. For example, the isotropic 3D unenhanced MR neurography (MRN) permits a detailed depiction of the extracranial peripheral branches for the CNs V, VII, and IX-XII. MRN aims to provide high-resolution images and uniform fat saturation and in the same time to suppress pulsation and breathing artifacts [48]. It is obtained using a 3D black-blood TSE STIR sequence that uses a pseudo steady-state sweep combined with a motion-sensitized driven equilibrium pulse [46,49,50]. Furthermore, the CNs tractography based on diffusion tensor imaging (DTI) may show promising results in tracing CNs thus supplementing the MRN anatomical diagnostic accuracy. At the moment, though only the thicker nerves and nerves as a group can be displayed by DTI [46, 48,51].

### Imaging findings

Detailed knowledge of normal CNs MRI appearance is essential for recognizing the abnormal features.

Gasserian ganglion and its three divisions are surrounded by a perineural vascular plexus (PNVP) down to their exit from the skull base and the plexus normally has mild thin contrast enhancement (CE), while the ganglion and its branches are avascular [52]. However, there are divergent reports about CE of the trigeminal ganglion: Williams et al. [52] obtained CE only in 4% of cases, while Downs et al. [53] found CE on all coronal T1-weighted spin-echo (SE) MRI, and later on, Yousry et al. [45] reported that the ganglion enhanced more than the adjacent dura on CE 3D TOF and on CE 3D CISS sequences. Meckel’s cave is filled with cerebrospinal fluid (CSF) and therefore it follows the CSF signal on all MRI sequences and has fluid density on CT. As stated above, the trigeminal ganglion that lies within the cave is thought to be avascular, so there should normally be no central CE in the Meckel’s cave [10].

The normal nerve is hypointense centrally and is incompletely (or sometimes completely) surrounded by an enhancing normal PNVP [52]. Therefore, the larger nerves (maxillary, mandibular, hypoglossal, and vidian) can be seen as an inner hypointense spot surrounded by hyperintense signal which appears as a “target sign” when sectioned perpendicularly or as a “tram-like” sign when imaged longitudinally. The thin CN VII, however, cannot be distinguished in the narrow facial canal from the PNVP [39]. Seemingly abnormal enhancement may occur due to head tilt or rotation. Subacute or asymptomatic neuritis can also cause nerve enhancement [52].

There is inhomogeneous, mild-to-moderate enhancement of the normal CN VII only in the facial canal (at least one segment may be enhanced), more evident in geniculate ganglion, proximal GSPN, tympanic and mastoid segment, but rarely in labyrinthine part [54,55]. An asymmetric CE is considered abnormal in these segments. In the cisternal, canalicular, and proximal extracranial segments, no enhancement was noticed and so CE here is always suspicious [54,55]. Gebarski et al. [54] motivated this fact with the presence of a rich circumneural arteriovenous plexus around the facial nerve in the temporal bone, which suddenly ends at the labyrinthine segment and the stylomastoid foramen. However, Martin-Duverneuil et al. [55] found also in healthy subjects intense enhancement, similar to pathological, in 6% of the geniculate ganglions and 11.6% of the tympanic segments. There may also be left-right asymmetry in CE [54]. Hong et al. [56], using 3D spoiled gradient recalled acquisition in steady state (FSPGR) at 3.0 T, demonstrated a variable extent of CE in all segments of the CN VII: mastoid – 100%; geniculate – 77.5%; tympanic – 37.5%; canalicular – 15%, and labyrinthine – 5%. Attention is needed when assessing the CN VII on newer sequences, as the study by Dehkharghani et al. [57] showed significantly higher signal intensity (SI) in most normal CN VII segments on unenhanced and contrast-enhanced 3D-FSPGR compared to SE.

Foramen ovale can have a different left-right size, but it is not normal when it varies by more than 4 mm on the two sides of a patient [43]. The right jugular foramen is larger than the left in 75% of the population [43].

The abnormal imaging features characteristic for PNS can be divided into primary – related to the structure modifications of the nerve and surrounding tissues, and secondary – alterations in the innervation territory of the affected nerve.

### Primary

**FIGURE 13:**
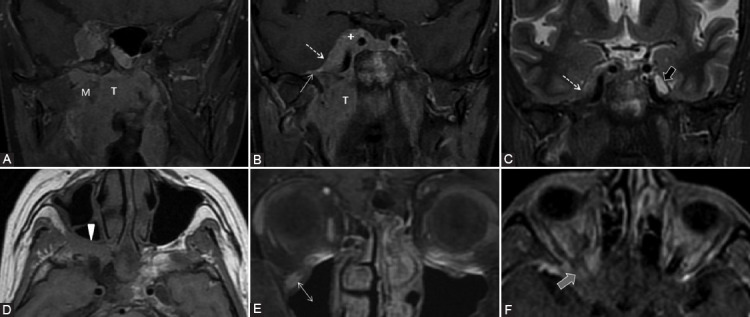
The right nasopharyngeal squamous cell carcinoma (T) in a patient with bilateral cervical adenopathy, right ptosis, and infraorbital skin paresthesia. Coronal contrast-enhanced T1-weighted fat saturated (A, B, E), coronal short-TI inversion recovery (C), axial T1 (D), and axial dynamic contrast-enhanced (F) images. There is tumor extension in the right masticator space (M) in the mandibular nerve distribution area, thus tumor cells spread retrograde to the foramen ovale (CN V3) which is enlarged (white arrow). Afterward, the neoplastic extension affects Meckel’s cave (dashed arrow) and cavernous sinus (+), with enlargement and CE. Notice the CSF signal in the normal left Meckel’s cave (black arrow) and it’s effacement on the right. Obliteration of the fat in the right PPF indicates CN V2 involvement (arrowhead). From here, there is anterograde PNS to the infraorbital nerve (double arrow). The right CN II invasion is also detected (gray arrow).

**FIGURE 14:**
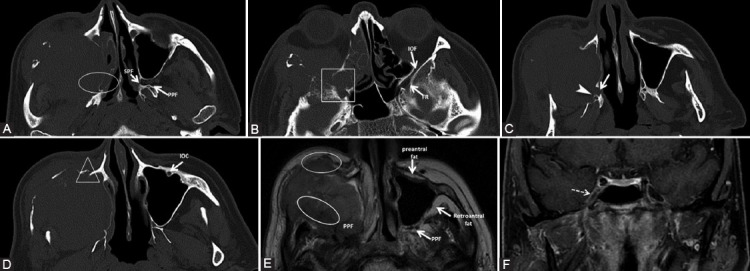
The patient presented with inflamed infraorbital and cheek region for about 3 weeks, no prior history of malignancy. Diagnosed squamous cell carcinoma of the maxillary sinus with orbital and ethmoidal extension. Bone window axial computed tomography scans (A, B, C, D) show direct tumor invasion of pterygopalatine fossa and sphenopalatine fissure (ellipse), as well as invasion of the inferior orbital fissure and foramen rotundum (rectangle). The tumor also invades the right hard palate with the enlargement of the greater (arrow) and lesser (arrowhead) palatine canals, and the infraorbital canal (triangle), compared to the normal left one. Axial T2-weighted image (E) shows the right PPF, pre- and retroantral fat obliteration (ellipses) versus the normal left side. T1-weighted fat-saturated coronal image (F) with contrast medium demonstrates the contrast enhancement of the right Meckel’s cave (dashed arrow), not seen on CT, suggesting the involvement of the trigeminal ganglion.

**FIGURE 15:**
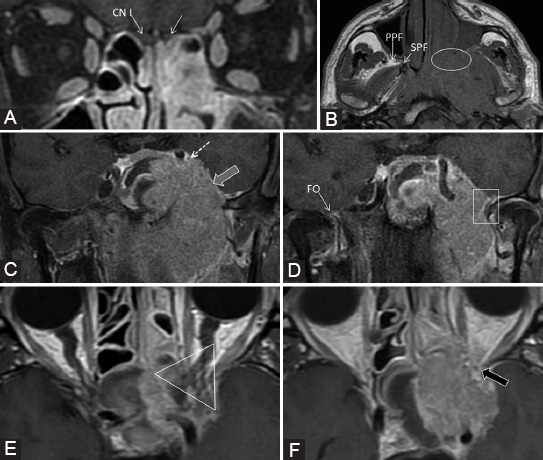
The left rhinopharyngeal squamous cell carcinoma with locoregional and skull base invasion. Coronal T1-weighted fat-saturated images (A, C, D); axial T1-weighted (B) and axial contrast-enhanced T1-weighted FSE (E, F) images. There is CE at the level of the left CN I (white arrow) which is invaded directly by the tumor (T). The PPF and sphenopalatine fissure (SPF) fat effacement (ellipse) confirms the involvement of V2 branch. There is enlargement and CE of the left Meckel’s cave (gray arrow) and cavernous sinus (dashed arrow), indicating the PNS along the CNs III, IV, V1, V2, and VI. Notice the foramen ovale invasion (CN V3, rectangle) compared to the right side (FO). The orbital apex together with the CN II is included in the tumor (triangle) and there is extension of pathologic tissue in the left superior orbital fissure (black arrow). Observe the slightly grayer appearance of CE (“evil gray”) on the non-fat-saturated sequences compared to the bright signal of fat. Notice the corresponding contralateral normal structures marked on the images.

**FIGURE 16:**
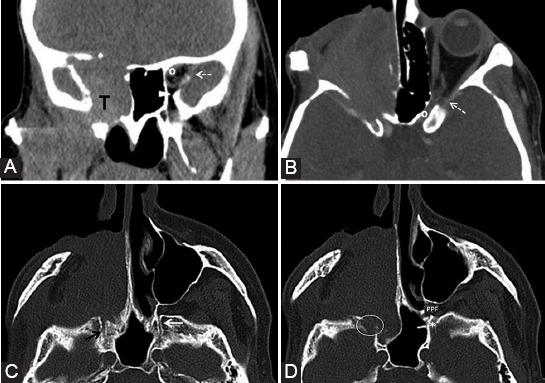
Second relapse of a neuroblastoma (T) of the right nasal fossa and maxillary sinus. Coronal reformatted computed tomography (CT) (A) and axial CT with contrast medium (B) images show complete invasion of the optic canal (o), superior (dashed arrow) and inferior orbital fissure (arrowhead) on the right compared to the normal left side (indicators for the normal left side). Axial CT scan (C, D, bone window) shows enlarged right vidian canal with slight blurring of its contour (black arrow) as compared to the left (white arrow). The right foramen rotundum (ellipse) is eroded compared to the left side (*). Notice the normal fat pad in the left pterygopalatine fossa (PPF) and total obliteration on the right.

1. Enhancement of a nerve associated with its enlargement and extension through the corresponding foramina is best visualized by MRI on any segment of the nerve (Figure 13). Pathologic enhancement is the diffuse enhancement with no clear separation between the nerve and its PNVP [24]. One must carefully assess the intensity, thickness, and right-left symmetry of CE to declare it pathological. On CT, even if the nerves cannot be well distinguished, the excessive enhancement in the neural foramina or canals can be viewed (Figure 9) and corresponds to the nerve’s involvement [8,10]. The enhancement could be explained by the disruption of the blood-nerve barrier as the tumor grows causing nerve damage [5,39];
2. Effacement or obliteration of the normal fat pads adjacent to neural foramina or in the PPF can be assessed both on MRI and CT (Figures 10 and 14) [5,10,56];
3. Widening or strong enhancement of the PPF, Meckel’s cave or cavernous sinus, can be visualized on MRI, as well as on CT [5,10]. The trigeminal cave is replaced with soft tissue and the cavernous sinus is enlarged, with bulging of the lateral dural margin [16] (Figure 15);
4. Foraminal or canal expansion or erosion due to nerve enlargement can be better observed as a side asymmetry on the bone CT scan [5,10] (Figures 10, 14 and 16). This is a tardive finding, since the normal nerve is smaller than the foramen and has sufficient time to grow until the bone destruction occurs. Attention should be paid, as the direct extension of the tumor may cause a similar appearance. The foraminal enlargement may be non-specific due to benign disease processes or anatomic variation, so the presence of erosions is more suggestive of a malignant etiology [8].

### Secondary

**FIGURE 17:**
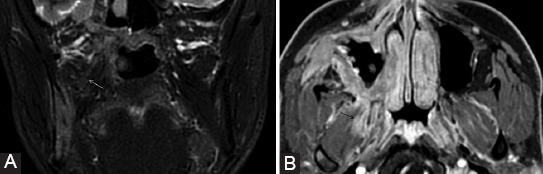
Same patient as in Figure 6. Coronal short-TI inversion recovery (A) shows right pterygoid muscle edema (white arrow). Axial contrast-enhanced 3D T1 FSPGR (B) reveals slight CE of the muscle (black arrow), compatible with acute muscular denervation suggesting the involvement of motor branches of V3.

1. Muscular denervation refers mainly to the muscles innervated by the mandibular nerve and undergoes three phases [7,59,60]:
  - Acute (<1 month) only seen on MRI, consists of T2 edema-like hypersignal and CE of the affected muscle with increased volume [7,60] (Figure 17). The similar signal to edema is due to a decrease in the intracellular water with increase in the extracellular water, which causes T2 prolongation [13,59]. The finding of CE may be due to an expanded extracellular space, with relatively increased vascularity and perfusion [59]. The denervated muscle preserves the internal striation, contrary to a muscular tumor infiltration which affects the muscular architecture [10];
  - Subacute (up to 12-20 months), also depicted on MRI, is the T2 prolongation, CE, no increase, or loss in volume, but additionally T1 hyperintensity due to fat transformation;
  - Chronic (after 12-20 months) changes consist of extensive fatty infiltration (T1 and T2 hyperintensity) associating neuropathic atrophy with volume loss which can also be observed on both CT and MRI [8,10]. In case of a direct tumor infiltration into the muscles, the volume of the affected muscular tissue often increases, the signal changes are not so generalized and the intensity is not as high as in chronic atrophy. In case of hypoglossal (CN XII) long-standing lesion, the affected hemi-tongue will show extensive fatty replacement and loss of tongue volume compared to the contralateral side, with possible prolapse of the involved hemi-tongue into the oropharynx [59].
    The muscles of facial expression (buccinators, orbicularis oculi, quadratus labii inferior, and platysma) also suffer denervation changes when CN VII is involved, seen on MRI merely as T2 hyperintensity and CE. Because of their small size, the potential volume loss is difficult to appreciate on imaging and a direct comparison from side to side is crucial [60].
2. Thickening and/or enhancement of the superior musculoaponeurotic system (SMAS)
  The SMAS layer represents a continuous fibrous network that connects the mimic muscles to the overlying dermis, as these muscles have their origin on bones but insert into dermis [61,62]. It has a fascial superficial layer, another one associated with the muscles of expressions and the deep layer attached to the periosteum. On CT, the SMAS is a hyperattenuating arcuate line, while on MRI, it is hypointense on both T1 and T2, being observed in the subcutaneous fat, deep to the skin, and superficial to the facial muscles, with no origin or insertion [61,63]. Related to imaging, SMAS can be defined as restricted to the facial region and blending with the parotid and temporoparietal fascia [63]. The peripheral CN VII branches (temporal, zygomatic, buccal, marginal mandibular, and cervical branches) exit the parotid gland and course deep to the SMAS before innervating the muscles of facial expression [61], while trigeminal sensory nerves lie superficial to SMAS. Therefore, one of the secondary imaging features to indicate PNS along CN VII branches is the nodular thickening and enhancement of the SMAS structures [8,40].
3. Rare indirect findings
  Small torus tubarius, mastoiditis, or middle ear effusion on the same side may indicate V3 denervation of the tensor veli palatini, causing Eustachian tube dysfunction [59].
  On routine non-focused, non-enhanced CT and MRI studies, the complete assessment of the CNs is difficult, but the obliteration of certain fat pads or chronic muscular denervation is usually visible and may indicate the presence of PNS. These can be the clues for the optimization of the imaging protocol for the specific CNs [40].
  Because the progression of PNS could be unpredictable, imaging should better be performed within 1 month before surgery [26].
  In the literature, a zonal classification system has been proposed by Williams et al., which may be useful for staging PNS regarding the extent of the anatomic spread, as it allows the surgeon to plan the optimal procedure to reach clear margins. In case of certain imaging evidence of PNS at the level of pre-pontine cistern (zone 3), the patient is considered probably inoperable [24,26]. The PNS can extend from the peripheral segments to central portions of CNs at the skull base up to the cisternal segments [24].

### Differential diagnosis, imaging pitfalls, and challenges

**FIGURE 18:**
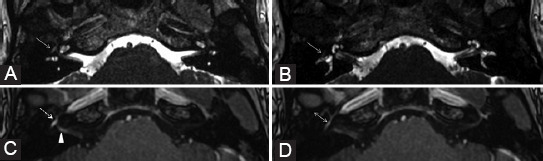
A 28-year-old patient with previous surgery for the right parotid pleomorphic adenoma (with facial nerve preservation) presents for the right peripheral facial nerve paralysis with rapid onset, biologically with inflammatory syndrome. Axial 3D FIESTA images (A, B) show a slight signal hyperintensity at the level of the tympanic segment (arrow) of the right CN VII. The contrast-enhanced 3D T1-weighted images (C, D) demonstrate moderate uniformly linear CE of the right geniculate ganglion (dashed arrow), canal (arrowhead), and tympanic (double arrow) segments, more intense than the normal left side. The symptoms improved after the treatment.

**FIGURE 19:**
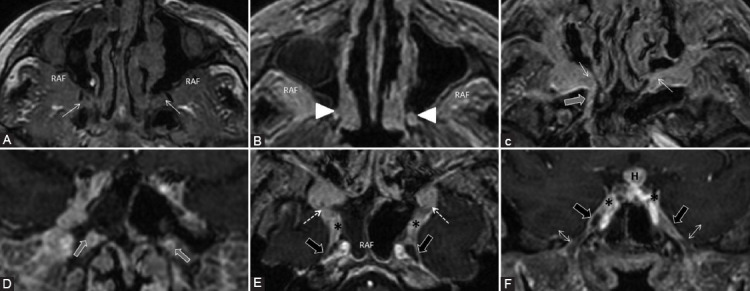
A 41-year-old male with IgG4-related disease with multiorgan manifestations: Central nervous system, orbital disease, sialadenitis, thoracic, and abdominal. Axial T1-weighted (A), axial (B, C, E), and coronal (D, F) contrast-enhanced 3D T1 images. Bilateral isointense infiltrative masses in the PPF (white arrows) and retroantral fat (RAF) are revealed, with corresponding homogeneous CE. There is enlargement and CE of great palatine (arrowheads) and vidian nerves (gray arrows). Enhancing tissue is observed also at the cavernous sinuses (*) extending to the rotundum foramen (dashed arrows), while Meckel’s cave appears normal (black arrows). The foramen ovale is also spared and normal hypointense CN V3 can be noticed surrounded by the contrast filled venous plexus (double arrow). Symmetric pachymeningeal thickening and enhancement are present. Notice the enhancement of the hypophysis (H).

**FIGURE 20:**
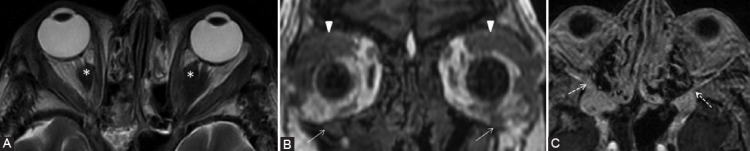
Same patient as in Figure 19. Axial T2-weighted (A), coronal T1-weighted (B), and axial contrast-enhanced 3D T1 (C) images. There are T2 hypointense focal masses (*) encasing both optic nerves. The infraorbital nerves are thickened, expand the infraorbital canal bilaterally (arrows), and show pathologic CE that extends to the inferior orbital fissure (dashed arrows) bilaterally. Involvement of the frontal nerves (branch of V1) is suggested by the bilateral fat obliteration (arrowheads) above the superior orbital levator muscle.

There are some neural pathologies that may exhibit abnormal neural enhancement or may occur adjacent to foramina and so can extend through, therefore, these entities should be differentiated from PNS, as they mimic it on imaging and may lead to false-positive results [5,10]. For example:

- Benign nerve tumors (neurofibromas and schwannomas) usually show bulky enlargement of the nerve portion when the patient already has symptoms. These also may extend beyond the neural foramina (meningioma);
- Neuritis is characterized by CE of the nerve, but no considerable increase in nerve diameter. It is caused by different infectious diseases such as viral neuritis (Figure 18) or mucormycosis, autoimmune conditions such as sarcoidosis, amyloidosis, or IgG disease (Figures 19 and 20), as well as postradiation.

Regarding all this imaging “mimickers,” the correlation with clinical history and the short-term follow-up scanning (at 6-8 weeks) or even biopsy when possible helps to establish the correct diagnosis [26]. Therefore, the PNS tends to progress, whereas neuritis improves and the benign tumors usually are relatively stable overtime [8].

Some imaging pitfalls can lead to misdiagnosis. The inadequate technique may lead to poor depiction of CNs and the main checkpoints, thus the lesions can be easily missed. Even in a clinical context of H and N neoplasms, some imaging findings may be very subtle at the early stages of the disease, and so, may resemble normal CNs appearance (discussed earlier). A thorough discussion with the multidisciplinary team as well as follow-up studies to assess progression is implied. The asymmetric scanning of the patient results in false asymmetry of the neural foramina. There are also anatomic variants such as the high-riding jugular bulb (more common on the right side) which may be associated with a large jugular fossa and may be interpreted as a sign of mass lesion on non-CE CT [43]. The imaging control following surgery or radiotherapy may resemble the initial appearance of PNS and last for a long time, therefore, it is very difficult to assess for local recurrence [10]. The radiation-induced neuropathy can appear as nerve thickening and CE and lead to false-positive findings. The only clue of the disease progression in this case is the development of new symptoms and advances in radiologic appearance on the serial follow-up imaging studies. In children and young adults, the bone marrow in the mandible is not fatty and enhances normally after contrast medium administration, so it makes the detection of potential PNS in the inferior alveolar canal very problematic [40]. Post-radiation myositis should be differentiated from neurotropic muscle atrophy [60]. Traumatized muscles showing T2 hyperintensity and CE may look similar to denervated muscles, but they are usually accompanied by subcutaneous edema and clinical context of trauma [60].

## CONCLUSION

An accurate clinical history, a solid neuroanatomical knowledge of CNs, their normal and pathologic appearance, an active search for the key radiologic landmarks, and an appropriate targeted technique (with the adaptation to the available possibilities in each specific imaging center) can provide a correct diagnosis. Given the impact of PNS on treatment strategies and prognosis, it is mandatory to describe this condition when staging patients with H and N cancers.
